# Essential Oil of *Symplocos chinensis* (Lour.) Druce: Chemical Composition, Antioxidant Activity, and Inhibitory Effects on Acetylcholinesterase and β-Lactamase

**DOI:** 10.3390/molecules31132372

**Published:** 2026-07-06

**Authors:** Zhuoyi Du, Yusen Zhang, Zetong Li, Xu Liu

**Affiliations:** 1 SDU-ANU Joint Science College, Shandong University, Weihai 264209, China; zhuoyi-du@outlook.com (Z.D.); yusenzhangsdu@outlook.com (Y.Z.); zetongli123@outlook.com (Z.L.); 2 Marine College, Shandong University, Weihai 264209, China

**Keywords:** *Symplocos chinensis* (Lour.) Druce, essential oil, chemical composition, biological activity

## Abstract

In traditional Chinese medicine, *Symplocos chinensis* (Lour.) Druce is a well-known herbal remedy prescribed to treat malaria, snakebites, and thermal injuries (burns and scalds). The objective of this study was to characterize the volatile profile of *S. chinensis* essential oil (EO) and evaluate its therapeutic potential through antioxidant, acetylcholinesterase (AChE), and β-lactamase inhibitory assays. The major components of *S. chinensis* EO included squalene (12.08%), octanol (7.01%), edulan III (6.81%), *n*-Hexadecanoic acid (6.81%), geranylacetone (4.80%), β-longipinene (4.60%), 2-methyl-1-octene (2.55%), (*E*, *E*)-2,4-decadienal (2.15%), and linalool (2.00%). Antioxidant evaluation revealed that the *S. chinensis* EO possessed relatively moderate radical scavenging properties, achieving 39.6% inhibition of DPPH radicals at 10 mg/mL, while its activity against ABTS radicals yielded an IC_50_ of 6.85 ± 1.97 mg/mL. Its reducing power, as determined by the FRAP assay, was further quantified at 175.50 ± 23.25 µmol/g. Furthermore, the EO exhibited inhibitory activities against AChE (IC_50_ = 149.40 ± 16.92 μg/mL) and β-lactamase (IC_50_ = 30.20 ± 0.84 μg/mL). These results demonstrate that *S. chinensis* EO exhibits antioxidant, AChE-inhibitory, and β-lactamase-inhibitory activities in vitro, warranting further comprehensive, systematic studies to evaluate its biological properties and potential applications.

## 1. Introduction

As representative natural products, essential oils (EOs) have been extensively applied across diverse fields, including food, medicine, and cosmetics [1]. EOs are regarded as the essence of plants and can display complex natural mixtures of volatile compounds. Owing to their chemically diverse compositions, EOs typically exhibit a broad spectrum of pharmacological properties, demonstrating a notable ability to interact with multiple molecular targets and modulate biological processes at both cellular and multicellular levels [2]. EOs usually exhibit multiple bioactivities, such as antibacterial, antifungal, and antioxidant properties, as demonstrated by previous research [3].

Free radicals—reactive species characterized by the presence of one or more unpaired electrons at the atomic or molecular level—exert considerable influence on human physiological well-being [4]. Oxidative stress arises from a disruption of the oxidant-antioxidant equilibrium, leading to excessive free radical production and irreversible deterioration of essential biological constituents such as DNA, membrane lipids, and functional proteins, and this process could ultimately cause cardiovascular diseases such as myocardial infarction (MI) and heart failure [4]. Additionally, oxidative stress has been widely acknowledged as a key contributor to the pathogenesis of Alzheimer’s disease (AD) [5]. Consequently, EOs exhibiting antioxidant activity are widely considered promising natural antioxidant candidates.

Alzheimer’s disease (AD) is an age-related neurodegenerative condition predominantly affecting the elderly, characterized by progressive cognitive impairment and the aberrant deposition of beta-amyloid peptide (Aβ) [6]. The pathogenesis of AD involves multiple hypotheses, with oxidative stress and diminished acetylcholine levels as potential key pathologies [7,8]. The level of reactive oxygen species (ROS) increases due to the abnormal accumulation of Aβ, tau protein, and heme. Excessive ROS can damage essential cellular components, ultimately leading to impaired neural function and tissue dysfunction [9]. Therefore, the development of antioxidant-based therapeutic agents represents an increasingly essential area of research for treating AD [10]. Additionally, acetylcholine levels are comparatively reduced in patients with AD [11]. Acetylcholinesterase (AChE), responsible for the hydrolysis of acetylcholine, has been shown to promote the aggregation of Aβ peptides into Alzheimer-associated amyloid deposits, consequently amplifying their neurotoxic effects [12]. AChE inhibitors can suppress enzyme activity, thereby elevating acetylcholine levels and producing therapeutic effects [13]. Consequently, EO with antioxidant and anti-AChE properties may contribute to the discovery of novel candidates for AD-related studies.

β-Lactam antibiotics have been extensively employed in the treatment of bacterial infections, owing to their broad-spectrum antimicrobial efficacy, favorable tolerability, and comparatively low toxicity profile [14]. Nevertheless, the overuse of β-lactam antibiotics has accelerated the emergence and spread of bacterial resistance [15]. Among complex resistance mechanisms, enzyme-mediated resistance has emerged as a particularly alarming challenge. β-Lactamase, mainly produced by Gram-negative bacteria, can hydrolyze the β-lactam ring of β-lactam antibiotics, ultimately rendering the antibiotic ineffective [16]. Those EOs, consequently, possess anti-β-lactamase activity and may represent promising sources of bioactive compounds for combating antibiotic resistance.

**Figure 1:**
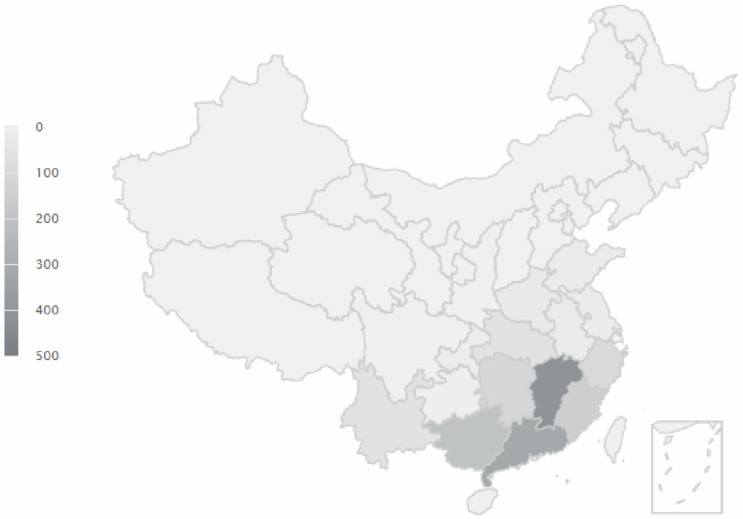
Distribution heatmap of *Symplocos chinensis* (Lour.) Druce in China based on specimen occurrence records retrieved from the Chinese Virtual Herbarium (CVH; https://www.cvh.ac.cn, accessed on 2 July 2026).

The *Symplocos* (Symplocaceae) comprises approximately 300 species, mainly distributed in tropical and subtropical regions of Asia, Oceania, and the Americas [17] (pp. 75–76). *Symplocos* plants have significant medicinal value in traditional medicine. *Symplocos chinensis* (Lour.) Druce belongs to a shrub distributed in Jiangxi, Fujian, Guangdong, and Guangxi provinces of China (Figure 1), characterized by its elliptic leaves, which are covered with dense, grayish-yellow, crisped, and soft trichomes on branches and foliage. Conventionally, its roots have been used to treat malaria and acute nephritis, while the fresh juice from its leaves is administered orally with alcohol as an antidote for snakebites. Additionally, the crushed leaves are traditionally applied to treat burns and traumatic bleeding [17] (pp. 75–76). However, traditional medicinal applications primarily involve using the whole roots or leaves of *S. chinensis.* In contrast, the chemical composition and biological activities of the EO derived from the plant remain insufficiently characterized. To address this gap, the present study aimed to elucidate the chemical profile of *S. chinensis* EO and evaluate its antioxidant, anti-AChE, and anti-β-lactamase activities.

## 2. Results and Discussion

### 2.1. EO Chemical Component Analysis

**Figure 2:**
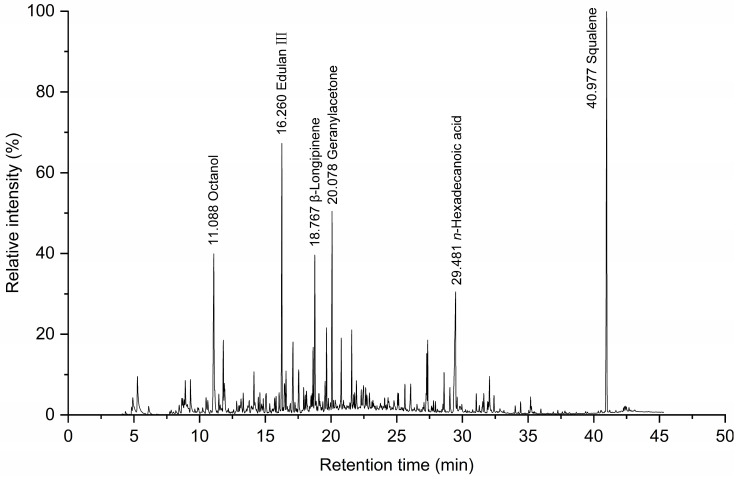
The total ion chromatogram (TIC) of *S. chinensis* essential oil (EO).

*S. chinensis* EO, a yellowish-green oil, was obtained by hydrodistillation from 828 g of dry plant material, which displayed a yield of 0.22 mL/kg. Figure 2 displays the total ion chromatogram (TIC) obtained from the essential oil (EO) of *S. chinensis*.

Using gas chromatography–mass spectrometry (GC-MS) (Supplementary Materials) combined with flame ionization detection (GC-FID), a total of 83 compounds were characterized, collectively representing 97.48% of the total composition (Table 1). The major constituents included squalene (12.08%), octanol (7.01%), edulan III (6.81%), *n*-Hexadecanoic acid (6.81%), geranylacetone (4.80%), β-longipinene (4.60%), 2-methyl-1-octene (2.55%), (*E*, *E*)-2,4-decadienal (2.15%), and linalool (2.00%). Further classification revealed that aliphatic compounds accounted for the majority (63.52%), followed by terpenoids (30.21%) and aromatic compounds (3.35%).

Squalene (12.08%), the most abundant compound in *S. chinensis* EO, exhibited multiple properties typical of a triterpenoid, including immunity enhancement, anti-skin aging, hypolipidemic, antioxidant, antitumor, antibacterial, and detoxifying effects. Therefore, it contributes substantially to the food and pharmaceutical industries [18]. The second most abundant compound, octanol (7.01%), demonstrated potent antifungal activity against *Botrytis cinerea*, a pathogenic fungus that causes grey mould [19]. In addition, plant-derived octanol exhibited broad-spectrum antimicrobial activity and food safety advantages [20,21]. As a consequence, this compound may serve as a natural and effective fungicide. *n*-Hexadecanoic acid (6.81%) exhibited antioxidant and antitumor properties. As a result, this compound has been recognized as a potential agent for therapeutic interventions in conditions including cancer [22,23]. Furthermore, geranylacetone (4.80%) has been shown to exhibit repellent activity against aphids, a pest group responsible for devastating economic losses in temperate-climate agriculture [24], indicating its potential as an antifeedant against these insects. Considering the considerable phytochemical diversity identified in *S. chinensis* EO, comprehensive in vitro investigations were undertaken to explore its antioxidant efficacy and inhibitory effects on key enzymes.

### 2.2. Antioxidant Activity Evaluation

**Figure 3:**
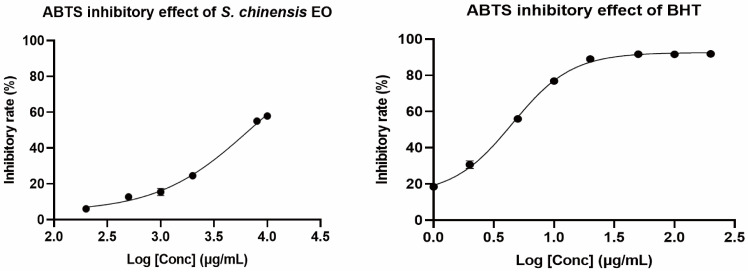
ABTS activity of *S. chinensis* EO and BHT at different concentrations.

To comprehensively evaluate the antioxidant potential of the samples, several analytical methods were employed in this study. At the maximum tested concentration of 10 mg/mL, the DPPH radical inhibition rate reached only 39.6%. In contrast, the IC_50_ of the positive control BHT was 52.33 ± 1.05 μg/mL (Table 2), suggesting that the essential oil (EO) exhibited a relatively limited capacity to scavenge DPPH radicals. Regarding ABTS radical scavenging activity, as illustrated in Figure 3, the activity of *S. chinensis* EO displayed a characteristic S-shaped dose–response curve, with an IC_50_ value of 6.85 ± 1.97 mg/mL (Table 2), reflecting a moderate level of ABTS radical scavenging ability.

The total antioxidant capacity of the EO was further evaluated using the FRAP assay. In contrast to free radical scavenging methods, this assay measures the overall reducing power of a sample by monitoring the electron transfer-mediated reduction of Fe^3+^ to Fe^2+^ under acidic conditions, which produces a characteristic color shift from yellow to bluish-green. The magnitude of this color change is directly proportional to the sample’s reductive capacity, thus reflecting its antioxidant potential [25]. As presented in Table 2, the *S. chinensis* EO exhibited a FRAP value of 175.50 ± 23.25 µmol/g (Trolox equivalent antioxidant concentration), demonstrating moderate overall antioxidant activity.

The three phenyl rings in DPPH create steric effects that hinder hydrogen atom transfer [26]. In contrast, the ABTS method operates through an electron transfer mechanism, rendering it applicable to both hydrophilic and lipophilic antioxidants [26]. Accordingly, the EO demonstrated moderately notable ABTS scavenging capacity alongside limited DPPH radical scavenging capacity. The FRAP assay further reflected the moderate total reducing power of *S. chinensis* EO. A higher degree of conjugation is generally associated with enhanced antioxidant performance [27]. Based on compositional analysis, *S. chinensis* EO is particularly rich in conjugated systems, encompassing terpenoids such as squalene (12.08%), linalool (2.00%), and isoshyobunone (1.70%), as well as α,β-unsaturated carbonyl compounds including (*E*, *E*)-2,4-decadienal (2.15%) and damascenone (1.57%). Therefore, the moderate antioxidant activity of *S. chinensis* EO is likely driven by its abundant conjugated terpenoids and α,β-unsaturated carbonyl constituents.

### 2.3. Anti-AChE Capacity of S. chinensis EO

Alzheimer’s disease (AD) ranks among the most widespread chronic brain-degenerative conditions worldwide. The enzyme acetylcholinesterase (AChE) plays a pivotal role in its pathophysiology, and targeting this enzyme through inhibition has emerged as a well-established therapeutic strategy [28]. Based on this, the AChE inhibitory activity of *S. chinensis* EO was evaluated.

**Figure 4:**
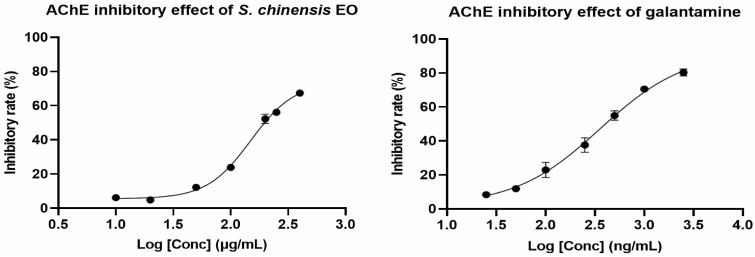
AChE inhibitory activities of *S. chinensis* EO and galantamine at varying concentrations.

According to the tests, *S. chinensis* EO inhibited AChE with an IC_50_ value of 149.40 ± 16.92 μg/mL, whereas galantamine (positive control) yielded an IC_50_ of 357.0 ± 52.82 ng/mL (Table 3). The concentration-dependent inhibitory profiles are displayed in Figure 4. The AChE inhibitory activity of *S. chinensis* EO was weaker than that of the positive control. For the purpose of determining the relative efficacy of the EO, a comparative analysis was conducted between its AChE inhibitory activity and that of other plant-derived EOs. In comparison, *S. chinensis* EO displayed stronger inhibitory activity than EO from *Coriandrum sativum*, *Carum carvi*, and *Arbutus unedo* (IC_50_ = 0.68 ± 0.03 mg/mL, 0.82 ± 0.05 mg/mL, 378.57 ± 0.05 μg/mL) [29,30]. *Pulicaria undulata* EO (IC_50_ = 139.30 ± 2.00 μg/mL) exhibited comparable inhibitory activity to *S. chinensis* EO [31]. Overall, these results indicated that *S. chinensis* EO exhibited moderate AChE-inhibitory activity.

Squalene (12.08%) was the most abundant compound identified in *S. chinensis* EO. Studies have shown that squalene exhibits considerable anti-AChE capacity, achieving an inhibition rate exceeding 80% at 100 μg/mL [32]. Therefore, squalene may serve as a key contributor to the observed AChE inhibitory effect of the EO. An in vivo study also showed that linalool (2.00%) inhibits oxidative stress-induced increases in AChE activity via the Nrf2/HO-1 pathway [33]. Therefore, despite the in vitro experiment of our EO assays, linalool could potentially contribute to the observed anti-AChE activity. Other monoterpene alcohols, α-terpineol (1.17%) and terpinen-4-ol, have also been documented to inhibit AChE (IC_50_ = 1.30 ± 0.06, 3.20 ± 2.30 mg/mL) [34]. These compounds may therefore serve as minor contributors to the observed anti-AChE activity of the EO. It should be noted that the discussion of individual constituents is based on previous studies and is presented for illustrative purposes only. Their actual contributions and possible synergistic or antagonistic interactions remain to be determined by further investigation.

### 2.4. Anti-β-Lactamase Capacity of S. chinensis EO

**Figure 5:**
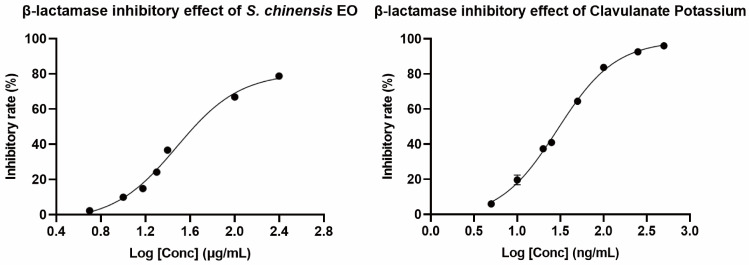
β-Lactamase inhibitory activities of *S. chinensis* EO and clavulanate potassium.

The growing prevalence of antibiotic resistance has emerged as a critical public health challenge. β-lactamases hydrolyze the amide bond within the β-lactam ring, thereby rendering β-lactam antibiotics ineffective [35]. One promising approach to overcoming this resistance mechanism involves the administration of β-lactamase inhibitors [36]. Based on this fact, the present work examined the anti-β-lactamase activity of *S. chinensis* EO. The inhibitory sigmoid curves are displayed in Figure 5. As shown in Table 3, the IC_50_ value was 30.20 ± 0.84 µg/mL, while the IC_50_ for clavulanate potassium was 29.23 ± 1.83 ng/mL. The inhibitory activity of the positive control was much stronger than that of the *S. chinensis* EO. Compared with previous studies, *S. chinensis* EO exhibits stronger inhibitory activity than EOs from *Clerodendrum fortunatum* L. and *Clerodendrum cyrtophyllum* Turcz. (IC_50_ = 673.50 ± 1.27, 41.34 ± 0.84 μg/mL) [37]. Therefore, *S. chinensis* EO demonstrates modest anti-β-lactamase activity. Previous papers have indicated that certain terpenoid compounds, such as α-terpineol, linalool, and citral, displayed comparatively superior inhibitory activity against β-lactamase [38]. These compounds were present in relatively high abundance in *S. chinensis* EO. Therefore, we preliminarily hypothesize that the monoterpenoid compounds present in abundant content may be associated with the modest anti-β-lactamase activity of *S. chinensis* EO.

## 3. Materials and Methods

### 3.1. Plant Material

Fresh specimens of *S. chinensis* were collected in September 2024 from Qinbei District, Qinzhou City, Guangxi, China (22.0232° N, 108.6562° E). The species was authenticated as *Symplocos chinensis* (Lour.) Druce by Prof. Hong Zhao through morphological examination. A voucher specimen (accession no. EO2408) has been deposited at the Biological Science Analysis and Testing Center, Shandong University, Weihai, China.

### 3.2. EO Extraction

The aerial portions of *S. chinensis* (828 g) were ground into fine particles and transferred into a 5 L round-bottomed flask containing 2.5 L of ultrapure water. Essential oil was obtained through hydrodistillation over approximately 4 h using a Clevenger-type apparatus (Synthware, Chongqing, China), following the procedure described in a previous study [39]. The oil fraction was subsequently separated from the aqueous phase by diethyl ether extraction. The resulting essential oil was dried over anhydrous sodium sulfate to remove residual moisture, and the solvent was then carefully evaporated under a stream of nitrogen gas in a dark, sealed environment.

### 3.3. The Chemical Constituent Analysis

The chemical composition and relative content of the EO were determined by GC-MS and GC-FID analysis [26]. GC-MS analysis was carried out using an Agilent 7890 GC coupled with a 5975C mass detector (Agilent Technologies, Santa Clara, CA, USA) fitted with an HP-5MS column (30 m × 0.25 mm, 0.25 μm). The oven temperature was programmed from 50 °C (4 min) to 280 °C at 6 °C/min and held for 3 min. Helium (99.999%) was used as the carrier gas at 1.0 mL/min. The injector, transfer line, and quadrupole temperatures were maintained at 260, 280, and 150 °C, respectively. Mass spectra were acquired in EI mode (70 eV) within the *m*/*z* range of 25–500. Samples were prepared in dichloromethane (1%, *w*/*v*), and 0.3 μL was injected without splitting. Relative quantification of the constituents was obtained by GC-FID using a PerkinElmer Clarus 500 instrument (Shelton, CT, USA) equipped with an HP-5 column of identical dimensions (Agilent, Santa Clara, CA, USA). The chromatographic conditions were the same as those applied in GC-MS analysis. Nitrogen was employed as the carrier gas (1.1 mL/min), and the injector and detector temperatures were set at 260 and 305 °C, respectively.

Component identification was achieved by matching mass spectra with the NIST/EPA/NIH 2023 database, with further confirmation through comparison of retention indices against published reference values. Retention indices were calculated using a series of *n*-alkane standards (C_8_–C_30_) on an HP-5MS capillary column.

### 3.4. Antioxidant Activities Evaluation

#### 3.4.1. DPPH Method

The free radical scavenging activity was evaluated using the 2,2-diphenyl-1-picrylhydrazyl (DPPH) assay, performed with slight modifications to a previously established method. Butylated hydroxytoluene (BHT) was used as the positive control. Stock solutions of BHT, essential oil (EO), and DPPH were prepared in ethanol at concentrations of 1.0 mg/mL, 50 mg/mL, and 0.17 mmol/L, respectively. In each assay, 50 μL of the EO solution and 200 μL of the DPPH solution were pipetted into individual wells of a 96-well microplate. The mixture was then allowed to react for 30 min at 25 °C under dark conditions, after which the absorbance was measured at 516 nm using an Epoch microplate spectrophotometer (BioTek Instruments, Minneapolis, MN, USA) [40]. All measurements were carried out in triplicate to ensure reproducibility. The DPPH radical scavenging capacity (RSC%) was calculated according to Formula (1):

(1) $$RSC\%=(1-\frac{A_{Sample}-A_{Sample\;blank}}{A_{Control}})\times 100\%$$

where *A_Sample_* represents the absorbance measured from the sample at each concentration, *A_Control_* refers to the absorbance of the DPPH control solution, and *A_Sample blank_* indicates the absorbance of the ethanol blank sample in the absence of DPPH.

#### 3.4.2. ABTS Method

The ABTS radical scavenging assay was performed following a previously reported protocol with minor modifications [40]. Stock solutions of butylated hydroxytoluene (BHT, used as a positive control) and the essential oil (EO) were separately prepared in anhydrous ethanol across a series of concentrations—1.0, 2.0, 5.0, 10.0, 20.0, 50.0, 100.0, and 200.0 μg/mL for BHT, and 0.2, 0.5, 1.0, 2.0, 8.0, and 10.0 mg/mL for EO. For the assay, 50 µL of each serially diluted EO solution in ethanol was combined with 200 µL of the pre-prepared ABTS^+•^ working solution in a microplate well. The mixture was briefly agitated for 10 s to achieve homogeneity, then allowed to react at room temperature for 6 min. Absorbance was subsequently recorded at 734 nm. The radical scavenging capacity (RSC%) of ABTS was determined using Equation (2) to assess the antioxidant potential of the samples:

(2) $$RSC\%=\frac{A_{0}-A}{A_{0}}\times 100\%$$

where *A*_0_ denotes the absorbance measured at 734 nm for the blank mixture consisting of 200 µL of ABTS^+•^ working reagent combined with 50 µL of pure ethanol, and A refers to the corresponding absorbance recorded when the ethanol was replaced by an equal volume (50 µL) of the EO sample solution. The IC_50_ value was determined by fitting the data to a sigmoidal dose–response curve using GraphPad Prism 9.5.

#### 3.4.3. FRAP Method

The FRAP assay was performed following a previously reported method [26]. A calibration curve constructed with Trolox served as the reference standard, and the total antioxidant capacity of the EO was expressed as Trolox equivalents. Stock solutions of EO were prepared at 50 mg/mL in ethanol and subsequently diluted to working concentrations of 0.1, 0.2, 1.0, 2.0, and 5.0 mg/mL. In a microplate format, 50 μL of each EO concentration was mixed with 200 μL of freshly prepared FRAP reagent, followed by incubation at 37 °C under light-shielded conditions. Absorbance was then measured at 593 nm. The antioxidant activity equivalent to Trolox was determined by substituting the measured absorbance (A) of a known sample concentration into the calibration equation. All measurements were conducted in triplicate, and results are reported as mean values.

### 3.5. Anti-AChE Activity Test

Galanthamine served as the positive control inhibitor in this assay [41]. The essential oil (EO) was diluted with phosphate-buffered saline (PBS, pH 8.0) to prepare a series of working concentrations: 400, 250, 200, 100, 50, 20, and 10 μg/mL. For each reaction, 25 μL of the EO sample was added to a well pre-loaded with 75 μL of PBS (pH 8.0) and 80 μL of acetylcholinesterase (AChE) solution (0.28 U/mL). The plate was then incubated in the dark for 20 min. 5,5′-Dithiobis-(2-nitrobenzoic acid) (DTNB) was used as the chromogenic reagent, and acetylthiocholine iodide (ATCI) was used as the substrate. Absorbance was recorded at 412 nm, and the enzymatic reaction rate was determined from the absorbance–time curve. Blank control measurements were conducted under identical conditions, with the EO solution replaced by 20 μL of solvent. The AChE inhibitory rate was calculated according to the following equation:

(3) $$Inhibition\%=\frac{K_{0}-K}{K_{0}}\times 100\%$$

where *K* and *K*_0_ represent the reaction rate measured in the presence and absence of the test sample, respectively, the IC_50_ value was fitted through an S-shaped curve using GraphPad Prism 9.5.

### 3.6. Anti-β-Lactamase Activity Test

Potassium clavulanate was used as the positive control inhibitor. The EO was prepared as a serial dilution in phosphate-buffered saline (PBS, pH 7.0) to yield working concentrations of 2.50, 1.00, 0.25, 0.20, 0.15, 0.10, and 0.05 mg/mL. For the assay, each well of a 96-well microplate received 20 µL of β-lactamase solution (5000 U/mL), 110 µL of PBS (50 mM, pH 7.0), and 20 µL of the EO sample. Following thorough mixing, the plate was incubated at 31 °C for 10 min, after which 50 µL of nitrocefin solution (0.1 mg/mL) was introduced into each well. Incubation was continued at 31 °C for an additional 10 min, and the optical density was subsequently measured at 489 nm [42]. The percentage inhibition was calculated according to Equation (4):

(4) $$Inhibition\%=(1-\frac{A_{s}-A_{sb}}{A_{e}-A_{b}})\times 100\%$$

where *A_s_* denotes the absorbance measured in the EO-treated well, *A_sb_* refers to the absorbance of the background well, *A_e_* corresponds to the absorbance obtained from the uninhibited reaction, and *A_b_* represents the absorbance of the blank control. IC_50_ values were determined by applying non-linear regression analysis.

### 3.7. Statistical Analysis

All experiments were performed in triplicate, and the results are expressed as mean ± standard deviation. IC_50_ values were calculated by nonlinear regression analysis using GraphPad Prism 9.0 (GraphPad Software, San Diego, CA, USA).

## 4. Conclusions

This study performed a comprehensive chemical characterization of *S. chinensis* essential oil (EO), leading to the identification of 83 distinct compounds. The predominant constituents were squalene (12.08%), octanol (7.01%), edulan III (6.81%), *n*-Hexadecanoic acid (6.81%), geranylacetone (4.80%), β-longipinene (4.60%), 2-methyl-1-octene (2.55%), (*E*, *E*)-2,4-decadienal (2.15%), and linalool (2.00%), collectively reflecting the remarkable chemical complexity of this EO. The EO displayed moderate antioxidant properties and inhibitory effects against acetylcholinesterase (AChE) and β-lactamase, indicating its potential as a source of natural antioxidants and bioactive compounds. The results may provide useful insights into the in vitro antioxidant, anti-AChE, and anti-β-lactamase activities of *S. chinensis* EO and support further investigations into its potential biological applications.

Nevertheless, the current findings are primarily derived from in vitro assays, which offer only preliminary, macroscopic insights into the biological activities of this EO. Future studies incorporating microbiological approaches are necessary to determine whether the EO can enhance antibiotic efficacy or reverse β-lactam resistance in bacterial systems. In addition, cytotoxicity evaluation and cell-based assays are important to establish the safety profile of *S. chinensis* EO. Accordingly, combining molecular docking simulations with rigorous in vivo experimental studies is strongly recommended for further development and application of *S. chinensis* EO.

## Figures and Tables

**Table 1:** Chemical constituents of the EO from *S. chinensis*.

No.	RT	Compound *	RI	RI_refer_	Area (%)	CAS ID or NIST
1	4.912	Leaf alcohol	867	863	1.26	928-96-1
2	5.271	2-Methyl-1-octene	880	874	2.55	4588-18-5
3	6.117	Heptanal	905	901	0.48	111-71-7
4	8.444	Morillol	984	980	0.29	3391-86-4
5	8.645	Sulcatone	991	986	0.41	110-93-0
6	8.719	2-Amylfuran	993	993	0.64	3777-69-3
7	8.836	Sulcatol	997	994	0.42	1569-60-4
8	8.910	3-Isobutyl-1-cyclohexene	1000	1001	1.31	4104-56-7
9	9.068	Octanal	1006	1003	0.42	124-13-0
10	9.301	(*E*, *E*)-2,4-Heptadienal	1015	1012	1.24	4313-03-5
11	10.485	Isocamphane	1056	1052	0.55	473-19-8
12	10.623	(*E*)-2-Octenal	1061	1060	0.42	2548-87-0
13	11.088	Octanol	1078	1070	7.01	111-87-5
14	11.459	p-Cymenene	1091	1090	0.54	1195-32-0
15	11.564	Ethyl Sorbate	1095	1093	0.27	2396-84-1
16	11.808	Linalool	1104	1099	2.00	78-70-6
17	11.882	Nonanal	1107	1104	1.33	124-19-6
18	12.823	1-Terpineol	1143	1137	0.36	586-82-3
19	13.151	p-Vinylanisole	1156	1156	0.65	637-69-4
20	13.320	(*E*)-2-Nonenal	1162	1162	0.51	18829-56-6
21	13.775	Terpinen-4-ol	1180	1177	0.47	562-74-3
22	14.134	α-Terpineol	1194	1189	1.17	98-55-5
23	14.208	Methyl salicylate	1197	1192	0.39	119-36-8
24	14.462	Decanal	1207	1206	0.38	112-31-2
25	14.716	1-p-Menthen-9-al	1218	1217	0.50	29548-14-9
26	14.843	β-Cyclocitral	1223	1220	0.34	432-25-7
27	15.065	cis-Carveol	1232	1229	0.88	1197-06-4
28	15.340	Neral	1244	1240	0.27	106-26-3
29	15.721	lemonol	1260	1260	0.53	624-15-7
30	15.816	(*E*)-2-Decenal	1264	1263	0.37	3913-81-3
31	16.049	α-Citral	1274	1270	0.45	141-27-5
32	16.260	Edulan III	1283	1285	6.81	72468-40-7
33	16.451	Dihydroedulan	1291	1293	1.13	72746-44-2
34	16.577	(*E**, **Z*)-2,4-Decadienal	1296	1295	0.97	25152-83-4
35	16.905	Undecanal	1311	1307	0.28	112-44-7
36	17.106	(*E*, *E*)-2,4-Decadienal	1320	1317	2.15	25152-84-5
37	17.254	Dimethylnonadienol	1326	1329	0.32	67845-50-5
38	17.540	Megastigma-4,6(*E*),8(*Z*)-triene	1339	1336	1.46	71186-24-8
39	17.921	Dehydro-ar-ionene	1356	1354	0.62	30364-38-6
40	18.132	2-Undecenal	1366	1367	0.92	2463-77-6
41	18.481	2-butyl-2-Octenal	1382	1378	0.61	13019-16-4
42	18.555	2-Norprezizene	1385	1382	0.45	384096
43	18.640	Damascenone	1389	1386	1.57	23726-93-4
44	18.767	β-Longipinene	1395	1401	4.60	41432-70-6
45	19.084	Dihydro-α-ionone	1410	1409	0.53	31499-72-6
46	19.549	α-Ionone	1433	1426	0.70	127-41-3
47	19.666	Nerylacetone	1438	1435	1.78	3879-26-3
48	19.793	6-Hydroxydihydrotheaspirane	1444	1446	0.19	57967-68-7
49	20.078	Geranylacetone	1457	1455	4.80	689-67-8
50	20.322	Cyclamal	1469	1464	0.25	103-95-7
51	20.776	β-Ionone	1491	1491	1.60	14901-07-6
52	21.58	Isoshyobunone	1531	1535	1.70	21698-46-4
53	21.802	(+)-Nerolidol	1544	1543	0.24	142-50-7
54	21.929	6-(3-Isopropenyl-3-methyl-1-cyclopropen-1-yl)-6-methyl-2-heptanone	1549	1551	0.69	69296-87-3
55	22.299	Nerolidol	1569	1564	0.42	7212-44-4
56	22.479	Dodecanoic acid	1577	1567	0.84	143-07-7
57	22.638	(-)-Spathulenol	1585	1577	0.47	77171-55-2
58	22.733	(-)-Globulol	1590	1591	0.40	489-41-8
59	22.923	Hexadecane	1599	1600	0.45	544-76-3
60	24.087	Bisabolol oxide II	1662	1656	0.35	26184-88-3
61	24.341	(*E*)-Tetradec-2-enal	1676	1673	0.61	51534-36-2
62	24.795	Heptadecane	1700	1700	0.48	629-78-7
63	25.081	Pentadecanal	1716	1715	0.48	2765-11-9
64	25.144	(2*Z*, 6*E*)-Farnesal	1720	1721	0.39	4380-32-9
65	25.620	(2*E*, 6*E*)-Farnesal	1747	1737	0.64	502-67-0
66	26.064	Tetradecanoic acid	1773	1768	0.91	544-63-8
67	27.291	(*E*, *E*)-Farnesyl acetate	1843	1843	1.76	4128-17-0
68	27.355	Hexahydrofarnesyl acetone	1848	1844	1.60	502-69-2
69	27.947	(*E*)-11-Hexadecen-1-ol	1882	1877	0.22	61301-56-2
70	28.518	Myristyl monoethoxylate	1917	1919	0.19	2136-70-1
71	28.603	Farnesyl acetone	1923	1918	0.93	1117-52-8
72	29.047	Isophytol	1951	1948	0.69	505-32-8
73	29.481	*n*-Hexadecanoic acid	1977	1968	6.81	57-10-3
74	29.957	2-(Pentadec-14-en-1-yl)furan	2007	1999	0.27	465695
75	31.057	Thunbergol	2079	2073	0.46	25269-17-4
76	31.543	γ-Palmitolactone	2111	2106	0.36	730-46-1
77	31.628	Phytol	2117	2114	0.54	150-86-7
78	31.987	Linoleic acid	2141	2132	0.28	60-33-3
79	32.061	9-Octadecenoic acid	2147	2144	1.08	2027-47-6
80	32.400	Octadecanoic acid	2169	2172	0.48	57-11-4
81	34.430	4,9,13,17-Tetramethyl-4,8,12,16-octadecatetraenal	2313	2302	0.26	56882-09-8
82	35.192	Octadecanamide	2369	2374	0.44	124-26-5
83	40.977	Squalene	2838	2927	12.08	111-02-4
	Terpenes				30.21	
	Aromatic compounds				3.35	
	Aliphatic compound				63.52	
	Total identified				97.48	

Peak area percentage was determined from the total ion chromatogram (TIC). * Compound identification was achieved through a combination of mass spectral matching against the NIST/EPA/NIH 2023 Mass Spectral Database, consultation of relevant published literature, and comparison of retention indices with those of authentic standards on the HP-5MS column. RI denotes the retention index experimentally determined on an HP-5MS column, while R_refer_ represents the retention index referenced from the NIST/EPA/NIH 2023 Mass Spectral Database.

**Table 2:** The antioxidant activities of *S. chinensis* EO using DPPH, ABTS, and FRAP assays.

Samples	DPPH (IC_50_)	ABTS (IC_50_)	FRAP Antioxidant Capacity
*S. chinensis* EO	>10 mg/mL (39.6%)	6.85 ± 1.97 mg/mL	175.50 ± 23.25 µmol/g
BHT	52.33 ± 1.05 μg/mL	4.51 ± 0.21 μg/mL	-

**Table 3:** The activities for AChE and β-lactamase inhibition assays.

Tested Samples	AChE (IC_50_)	β-Lactamase (IC_50_)
*S. chinensis* EO	149.40 ± 16.92 μg/mL	30.20 ± 0.84 μg/mL
Galantamine	357.0 ± 52.82 ng/mL	-
Clavulanate Potassium	-	29.23 ± 1.83 ng/mL

## Data Availability

Data is available on request from the authors.

## Supplementary Materials

The following supporting information can be downloaded at: https://www.mdpi.com/article/10.3390/molecules31132372/s1, GC-MS Analysis Report.

## Abbreviations

The following abbreviations are used in this manuscript:

ABTS	2,2′-azino-bis(3-ethylbenzothiazoline-6-sulfonic acid)
AChE	Acetylcholinesterase
AD	Alzheimer’s disease
ATCI	Acetylthiocholine iodide
Aβ	Beta-amyloid
BHT	Butylated hydroxytoluene
DPPH	2,2-diphenyl-1-picrylhydrazyl
DTNB	5,5′-dithiobis(2-nitrobenzoic acid)
EO	Essential oil
EOs	Essential oils
FRAP	Ferric reducing antioxidant power
GC-FID	Gas chromatography–flame ionization detector
GC-MS	Gas chromatography–mass spectrometry
IC_50_	Half-maximal inhibitory concentration
MI	Myocardial infarction
PBS	Phosphate-buffered saline
ROS	Reactive oxygen species
RSC%	Radical scavenging capacity
*S. chinensis*	*Symplocos chinensis* (Lour.) Druce
TIC	Total ion chromatogram
Trolox	(±)-6-Hydroxy-2,5,7,8-tetramethylchromane-2-carboxylic acid
