# Supplementary material for: Essential Oil of Symplocos chinensis (Lour.) Druce: Chemical Composition, Antioxidant Activity, and Inhibitory Effects on Acetylcholinesterase and β-Lactamase
Source: Molecules. 2026 Jul 6;31(13):2372. doi: 10.3390/molecules31132372 (PMC13362661; doi:10.3390/molecules31132372)
Supplement: Supplementary file 1 [file molecules-31-02372-s001.zip › molecules-4376240-supplementary.pdf]

# Analysis Report

Agilent | Trusted Answers

## Sample Information

| | | | |
|---|---|---|---|
| SampleName | E02408 | DataFilePath | D:\GCMS\E02408.D |
| SampleID | | AcqTime_Local | 2025/1/13 20:16:40 (UTC+08:00) |
| InstrumentName | GCMS-5975C | MethodPath_Acquisition | 2025做样测试.M |
| MSType | Q | Version_AcqSW | |
| InjectionVolume | 0 | IRMStatus | |
| SamplePosition | 10 | MethodPath_Analysis | D:\GCMS\E02408.D\Results\Qual\Version4\deanwhite 240410.m |
| PlatePosition | | TargetSourcePath | D:\MassHunter\Library\NIST20.L;D:\MassHunter\PCDL\Pesticide_Example.cdb;D:\MassHunter\PCDL\Test_AM_PCDL.cdb |
| Operator | MassHunter GC/MS Translator | ResultSummary | |

## Sample Chromatograms

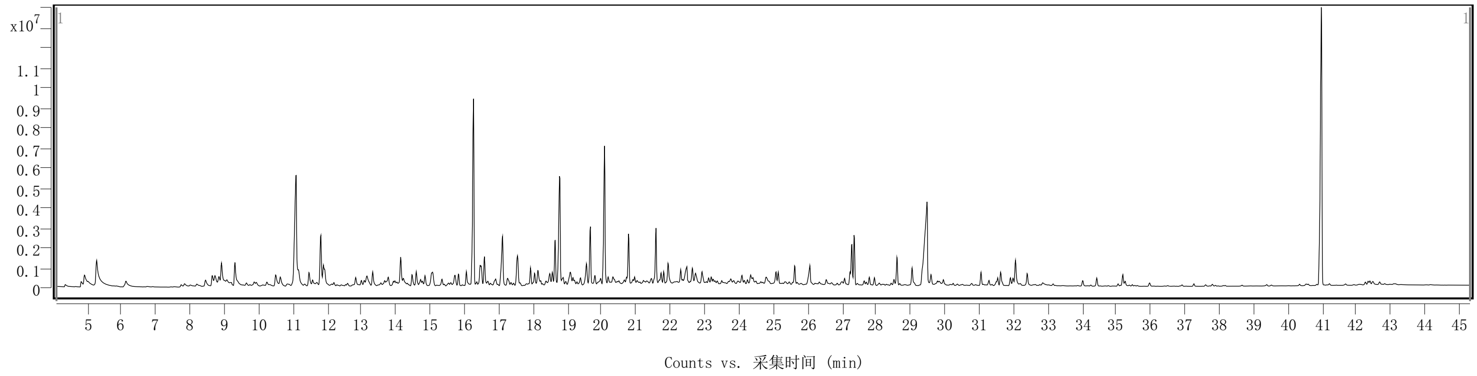


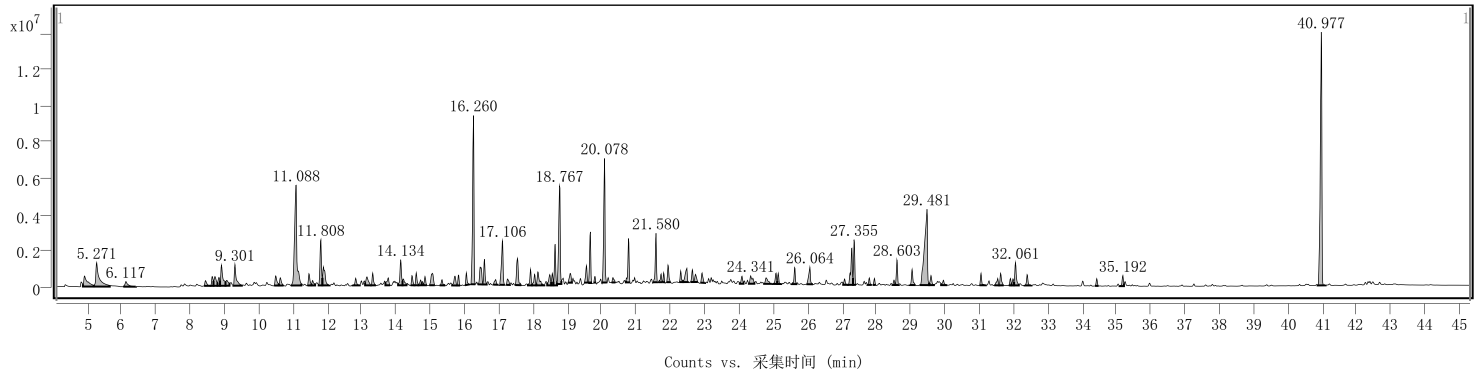


*Chromatogram Peaks*

| Peak | StartRT | RT | EndRT | Height | Area | AreaPct | SNR |
|---|---|---|---|---|---|---|---|
| 1 | 4.869 | 4.912 | 5.187 | 576997 | 4634053 | 10.40 | |
| 2 | 5.187 | 5.271 | 5.652 | 1315590 | 9414322 | 21.14 | |
| 3 | 6.045 | 6.117 | 6.414 | 278630 | 1787346 | 4.01 | |
| 4 | 8.403 | 8.444 | 8.592 | 285415 | 1085620 | 2.44 | |
| 5 | 8.592 | 8.645 | 8.677 | 507720 | 1510879 | 3.39 | |
| 6 | 8.677 | 8.719 | 8.793 | 508977 | 2372472 | 5.33 | |
| 7 | 8.793 | 8.836 | 8.867 | 458598 | 1560012 | 3.50 | |
| 8 | 8.867 | 8.910 | 9.026 | 1134136 | 4835386 | 10.86 | |
| 9 | 9.026 | 9.068 | 9.142 | 297328 | 1560672 | 3.50 | |
| 10 | 9.259 | 9.301 | 9.502 | 1160765 | 4588008 | 10.30 | |
| 11 | 10.445 | 10.485 | 10.581 | 531759 | 2020564 | 4.54 | |
| 12 | 10.581 | 10.623 | 10.724 | 425550 | 1565321 | 3.51 | |
| 13 | 10.930 | 11.088 | 11.236 | 5515356 | 25838112 | 58.01 | |
| 14 | 11.416 | 11.459 | 11.533 | 641630 | 2007563 | 4.51 | |
| 15 | 11.533 | 11.564 | 11.638 | 271993 | 981394 | 2.20 | |
| 16 | 11.744 | 11.808 | 11.850 | 2510318 | 7388798 | 16.59 | |
| 17 | 11.850 | 11.882 | 12.061 | 1013688 | 4908987 | 11.02 | |
| 18 | 12.738 | 12.823 | 12.939 | 394184 | 1326295 | 2.98 | |
| 19 | 13.098 | 13.151 | 13.267 | 466556 | 2382541 | 5.35 | |
| 20 | 13.267 | 13.320 | 13.413 | 671956 | 1883800 | 4.23 | |
| 21 | 13.690 | 13.775 | 13.827 | 409612 | 1741624 | 3.91 | |
| 22 | 14.060 | 14.134 | 14.187 | 1407751 | 4303779 | 9.66 | |
| 23 | 14.187 | 14.208 | 14.304 | 345932 | 1437930 | 3.23 | |
| 24 | 14.430 | 14.462 | 14.536 | 528341 | 1397246 | 3.14 | |
| 25 | 14.536 | 14.589 | 14.653 | 680891 | 1828774 | 4.11 | |
| 26 | 14.653 | 14.716 | 14.748 | 286999 | 924886 | 2.08 | |
| 27 | 14.801 | 14.843 | 14.899 | 474329 | 1270786 | 2.85 | |
| 28 | 14.970 | 15.065 | 15.129 | 651840 | 3237785 | 7.27 | |
| 29 | 15.268 | 15.340 | 15.432 | 309549 | 979941 | 2.20 | |
| 30 | 15.636 | 15.721 | 15.774 | 509208 | 1940614 | 4.36 | |
| 31 | 15.774 | 15.816 | 15.869 | 568315 | 1361258 | 3.06 | |
| 32 | 16.009 | 16.049 | 16.144 | 665299 | 1648475 | 3.70 | |
| 33 | 16.177 | 16.260 | 16.313 | 9285815 | 25087300 | 56.32 | |
| 34 | 16.377 | 16.451 | 16.525 | 949144 | 4147422 | 9.31 | |
| 35 | 16.525 | 16.577 | 16.641 | 1388166 | 3573321 | 8.02 | |
| 36 | 16.832 | 16.905 | 16.981 | 279681 | 1036054 | 2.33 | |
| 37 | 17.022 | 17.106 | 17.172 | 2419162 | 7920767 | 17.78 | |
| 38 | 17.213 | 17.254 | 17.318 | 327465 | 1173148 | 2.63 | |
| 39 | 17.473 | 17.540 | 17.652 | 1465169 | 5373764 | 12.06 | |
| 40 | 17.847 | 17.921 | 17.963 | 871610 | 2267741 | 5.09 | |

*Chromatogram Peaks*

| Peak | StartRT | RT | EndRT | Height | Area | AreaPct | SNR |
|---|---|---|---|---|---|---|---|
| 41 | 17.963 | 18.037 | 18.090 | 605933 | 1936366 | 4.35 | |
| 42 | 18.090 | 18.132 | 18.323 | 742125 | 3381924 | 7.59 | |
| 43 | 18.407 | 18.481 | 18.524 | 590635 | 2253464 | 5.06 | |
| 44 | 18.524 | 18.555 | 18.587 | 654316 | 1662222 | 3.73 | |
| 45 | 18.587 | 18.640 | 18.693 | 2266807 | 5803171 | 13.03 | |
| 46 | 18.693 | 18.767 | 18.820 | 5394051 | 16966453 | 38.09 | |
| 47 | 19.012 | 19.084 | 19.126 | 511582 | 1943006 | 4.36 | |
| 48 | 19.490 | 19.549 | 19.593 | 928755 | 2585809 | 5.81 | |
| 49 | 19.607 | 19.666 | 19.703 | 2786449 | 6553303 | 14.71 | |
| 50 | 19.763 | 19.793 | 19.827 | 332383 | 695766 | 1.56 | |
| 51 | 20.014 | 20.078 | 20.138 | 6827187 | 17708619 | 39.76 | |
| 52 | 20.283 | 20.322 | 20.397 | 265307 | 929073 | 2.09 | |
| 53 | 20.702 | 20.776 | 20.819 | 2417740 | 5909410 | 13.27 | |
| 54 | 21.500 | 21.580 | 21.621 | 2692847 | 6258981 | 14.05 | |
| 55 | 21.647 | 21.728 | 21.764 | 450366 | 1183203 | 2.66 | |
| 56 | 21.772 | 21.802 | 21.833 | 513167 | 890866 | 2.00 | |
| 57 | 21.884 | 21.929 | 22.004 | 929237 | 2538165 | 5.70 | |
| 58 | 22.260 | 22.299 | 22.373 | 610867 | 1558177 | 3.50 | |
| 59 | 22.373 | 22.479 | 22.528 | 753158 | 3096736 | 6.95 | |
| 60 | 22.585 | 22.638 | 22.680 | 678134 | 1737890 | 3.90 | |
| 61 | 22.680 | 22.733 | 22.799 | 422765 | 1484705 | 3.33 | |
| 62 | 22.878 | 22.923 | 23.008 | 535823 | 1640574 | 3.68 | |
| 63 | 24.023 | 24.087 | 24.129 | 414523 | 1272559 | 2.86 | |
| 64 | 24.288 | 24.341 | 24.446 | 432450 | 2242282 | 5.03 | |
| 65 | 24.732 | 24.795 | 24.954 | 327028 | 1774677 | 3.98 | |
| 66 | 24.986 | 25.081 | 25.113 | 596176 | 1769853 | 3.97 | |
| 67 | 25.113 | 25.144 | 25.229 | 588063 | 1441902 | 3.24 | |
| 68 | 25.546 | 25.620 | 25.673 | 909964 | 2344887 | 5.26 | |
| 69 | 25.966 | 26.064 | 26.155 | 928803 | 3337097 | 7.49 | |
| 70 | 27.037 | 27.080 | 27.175 | 319057 | 944512 | 2.12 | |
| 71 | 27.175 | 27.291 | 27.323 | 2018557 | 6483670 | 14.56 | |
| 72 | 27.323 | 27.355 | 27.408 | 2485567 | 5914068 | 13.28 | |
| 73 | 27.746 | 27.799 | 27.871 | 384407 | 930121 | 2.09 | |
| 74 | 27.904 | 27.947 | 27.986 | 356452 | 794936 | 1.78 | |
| 75 | 28.476 | 28.518 | 28.550 | 268211 | 684742 | 1.54 | |
| 76 | 28.550 | 28.603 | 28.656 | 1373328 | 3422587 | 7.68 | |
| 77 | 28.973 | 29.047 | 29.146 | 852785 | 2528091 | 5.68 | |
| 78 | 29.265 | 29.481 | 29.544 | 4170224 | 25089961 | 56.33 | |
| 79 | 29.544 | 29.597 | 29.682 | 520909 | 1718035 | 3.86 | |
| 80 | 29.893 | 29.957 | 30.071 | 271180 | 997714 | 2.24 | |
| 81 | 31.011 | 31.057 | 31.194 | 647065 | 1709370 | 3.84 | |
| 82 | 31.441 | 31.543 | 31.585 | 377177 | 1309168 | 2.94 | |
| 83 | 31.585 | 31.628 | 31.718 | 657497 | 1994132 | 4.48 | |
| 84 | 31.866 | 31.913 | 31.945 | 379593 | 941001 | 2.11 | |
| 85 | 31.945 | 31.987 | 32.019 | 360528 | 1039561 | 2.33 | |
| 86 | 32.019 | 32.061 | 32.156 | 1254369 | 3976120 | 8.93 | |
| 87 | 32.326 | 32.400 | 32.527 | 595798 | 1768562 | 3.97 | |
| 88 | 34.380 | 34.430 | 34.483 | 397841 | 969476 | 2.18 | |
| 89 | 35.097 | 35.192 | 35.224 | 581048 | 1635609 | 3.67 | |
| 90 | 40.871 | 40.977 | 41.104 | 13933734 | 44540587 | 100.00 | |

## Compound Summary

| Cpd | CpdName | Formula | RT | Mass | CAS | IDSourceBest | Score_ID | Score_Lib | Score_DB | Score_MFG | Algorithm |
|---|---|---|---|---|---|---|---|---|---|---|---|
| 1 | 3-Hexen-1-ol, (Z)- | C6 H12 O | 4.912 | | 928-96-1 | Lib searching | 92.35 | 92.35 | | | integral |
| 2 | 2-Methyl-1-octene | C9 H18 | 5.271 | | 4588-18-5 | Lib searching | 100.00 | | | | integral |
| 3 | Heptanal | C7 H14 O | 6.117 | | 111-71-7 | Lib searching | 83.95 | 83.95 | | | integral |
| 4 | 1-Octen-3-ol | C8 H16 O | 8.444 | | 3391-86-4 | Lib searching | 94.48 | 94.48 | | | integral |
| 5 | 5-Hepten-2-one, 6-methyl- | C8 H14 O | 8.645 | | 110-93-0 | Lib searching | 95.58 | 95.58 | | | integral |
| 6 | Furan, 2-pentyl- | C9 H14 O | 8.719 | | 3777-69-3 | Lib searching | 74.70 | 74.70 | | | integral |
| 7 | 5-Hepten-2-ol, 6-methyl- | C8 H16 O | 8.836 | | 1569-60-4 | Lib searching | 89.08 | 89.08 | | | integral |
| 8 | 3-Isobutyl-1-cyclohexene | C10 H18 | 8.910 | | 4104-56-7 | Lib searching | 100.00 | | | | integral |
| 9 | Octanal | C8 H16 O | 9.068 | | 124-13-0 | Lib searching | 100.00 | | | | integral |
| 10 | (E,E)-2,4-Heptadienal | C7 H10 O | 9.301 | | 4313-03-5 | Lib searching | 100.00 | | | | integral |
| 11 | 2,2,3-trimethyl-Bicyclo[2.2.1]heptane | C10 H18 | 10.485 | | 473-19-8 | Lib searching | 100.00 | | | | integral |
| 12 | 2-Octenal, (E)- | C8 H14 O | 10.623 | | 2548-87-0 | Lib searching | 84.65 | 84.65 | | | integral |
| 13 | 1-Octanol | C8 H18 O | 11.088 | | 111-87-5 | Lib searching | 97.45 | 97.45 | | | integral |
| 14 | 1-methyl-4-(1-methylethenyl)-Benzene | C10 H12 | 11.459 | | 1195-32-0 | Lib searching | 100.00 | | | | integral |
| 15 | (2E,4E)-2,4-Hexadienoic acid, ethyl ester | C8 H12 O2 | 11.564 | | 2396-84-1 | Lib searching | 100.00 | | | | integral |
| 16 | Linalool | C10 H18 O | 11.808 | | 78-70-6 | Lib searching | 98.85 | 98.85 | | | integral |
| 17 | Nonanal | C9 H18 O | 11.882 | | 124-19-6 | Lib searching | 80.96 | 80.96 | | | integral |
| 18 | 1-Terpinenol | C10 H18 O | 12.823 | | 586-82-3 | Lib searching | 100.00 | | | | integral |
| 19 | 1-ethenyl-4-methoxy-Benzene | C9 H10 O | 13.151 | | 637-69-4 | Lib searching | 100.00 | | | | integral |
| 20 | (E)-2-Nonenal | C9 H16 O | 13.320 | | 18829-56-6 | Lib searching | 100.00 | | | | integral |
| 21 | Terpinen-4-ol | C10 H18 O | 13.775 | | 562-74-3 | Lib searching | 60.50 | 60.50 | | | integral |
| 22 | .alpha.-Terpineol | C10 H18 O | 14.134 | | 98-55-5 | Lib searching | 94.73 | 94.73 | | | integral |
| 23 | Methyl salicylate | C8 H8 O3 | 14.208 | | 119-36-8 | Lib searching | 56.33 | 56.33 | | | integral |
| 24 | Decanal | C10 H20 O | 14.462 | | 112-31-2 | Lib searching | 100.00 | | | | integral |
| 25 | TMD (Deleted) | | 14.589 | | 30316-36-0 | Lib searching | 100.00 | | | | integral |
| 26 | α,4-dimethyl-3-Cyclohexene-1-acetaldehyde | C10 H16 O | 14.716 | | 29548-14-9 | Lib searching | 100.00 | | | | integral |
| 27 | 1-Cyclohexene-1-carboxaldehyde, 2,6,6-trimethyl- | C10 H16 O | 14.843 | | 432-25-7 | Lib searching | 88.72 | 88.72 | | | integral |
| 28 | cis-Carveol | C10 H16 O | 15.065 | | 1197-06-4 | Lib searching | 100.00 | | | | integral |
| 29 | Neral | C10 H16 O | 15.340 | | 106-26-3 | Lib searching | 100.00 | | | | integral |
| 30 | lemonol | C10 H18 O | 15.721 | | 624-15-7 | Lib searching | 100.00 | | | | integral |

## Compound Summary

| Cpd | CpdName | Formula | RT | Mass | CAS | IDSourceBest | Score_ID | Score_Lib | Score_DB | Score_MFG | Algorithm |
|---|---|---|---|---|---|---|---|---|---|---|---|
| 31 | (E)-2-Decenal | C10 H18 O | 15.816 | | 3913-81-3 | Lib searching | 100.00 | | | | integral |
| 32 | 2,6-Octadienal, 3,7-dimethyl-, (E)- | C10 H16 O | 16.049 | | 141-27-5 | Lib searching | 95.64 | 95.64 | | | integral |
| 33 | Edulan III | C13 H20 O | 16.260 | | 72468-40-7 | Lib searching | 100.00 | | | | integral |
| 34 | 2,5,5,8a-Tetramethyl-3,4,4a,5,6,8a-hexahydro-2H-chromene | C13 H22 O | 16.451 | | 72746-44-2 | Lib searching | 66.87 | 66.87 | | | integral |
| 35 | (E,Z)-2,4-Decadienal | C10 H16 O | 16.577 | | 25152-83-4 | Lib searching | 100.00 | | | | integral |
| 36 | Undecanal | C11 H22 O | 16.905 | | 112-44-7 | Lib searching | 100.00 | | | | integral |
| 37 | (E,E)-2,4-Decadienal | C10 H16 O | 17.106 | | 25152-84-5 | Lib searching | 100.00 | | | | integral |
| 38 | 4,8-dimethyl-3,7-Nonadien-2-ol | C11 H20 O | 17.254 | | 67845-50-5 | Lib searching | 100.00 | | | | integral |
| 39 | Megastigma-4,6(E),8(Z)-triene | C13 H20 | 17.540 | | 71186-24-8 | Lib searching | 100.00 | | | | integral |
| 40 | Naphthalene, 1,2-dihydro-1,1,6-trimethyl- | C13 H16 | 17.921 | | 30364-38-6 | Lib searching | 96.89 | 96.89 | | | integral |
| 41 | ? | | 18.037 | | | Lib searching | 100.00 | | | | integral |
| 42 | 2-Undecenal | C11 H20 O | 18.132 | | 2463-77-6 | Lib searching | 100.00 | | | | integral |
| 43 | 2-butyl-2-Octenal | C12 H22 O | 18.481 | | 13019-16-4 | Lib searching | 100.00 | | | | integral |
| 44 | 2-Norprezizene | C14 H22 | 18.555 | | | Lib searching | 100.00 | | | | integral |
| 45 | (E)-1-(2,6,6-trimethyl-1,3-cyclohexadien-1-yl)-2-Buten-1-one | C13 H18 O | 18.640 | | 23726-93-4 | Lib searching | 100.00 | | | | integral |
| 46 | β-Longipinene | C15 H24 | 18.767 | | 41432-70-6 | Lib searching | 100.00 | | | | integral |
| 47 | 4-(2,6,6-trimethyl-2-cyclohexen-1-yl)-2-Butanone | C13 H22 O | 19.084 | | 31499-72-6 | Lib searching | 100.00 | | | | integral |
| 48 | α-Ionone | C13 H20 O | 19.549 | | 127-41-3 | Lib searching | 100.00 | | | | integral |
| 49 | Nerylacetone | C13 H24 O2 | 19.666 | | 3879-26-3 | Lib searching | 100.00 | | | | integral |
| 50 | 6-Hydroxydihydrotheaspirane | C13 H24 O2 | 19.793 | | 57967-68-7 | Lib searching | 100.00 | | | | integral |
| 51 | 5,9-Undecadien-2-one, 6,10-dimethyl- | C13 H22 O | 20.078 | | 689-67-8 | Lib searching | 96.83 | 96.83 | | | integral |
| 52 | 3-(4-Isopropylphenyl)-2-methylpropionaldehyde | C13 H18 O | 20.322 | | 103-95-7 | Lib searching | 100.00 | | | | integral |
| 53 | 4-(2,6,6-trimethyl-1-cyclohexen-1-yl)-3-Buten-2-one | C13 H20 O | 20.776 | | 14901-07-6 | Lib searching | 100.00 | | | | integral |
| 54 | Isoshyobunone | C15 H24 O | 21.580 | | 21698-46-4 | Lib searching | 100.00 | | | | integral |
| 55 | ? | | 21.728 | | | Lib searching | 100.00 | | | | integral |
| 56 | Nerolidol | C15 H26 O | 21.802 | | 142-50-7 | Lib searching | 100.00 | | | | integral |
| 57 | 2-Heptanone, 6-methyl-6-[3-methyl-3-(1-methylethenyl)-1-cyclopropen-1-yl]- | C15 H24 O | 21.929 | | 69296-87-3 | Lib searching | 100.00 | | | | integral |
| 58 | 1,6,10-Dodecatrien-3-ol, 3,7,11-trimethyl- | C15 H26 O | 22.299 | | 7212-44-4 | Lib searching | 87.35 | 87.35 | | | integral |
| 59 | Dodecanoic acid | C12 H24 O2 | 22.479 | | 143-07-7 | Lib searching | 100.00 | | | | integral |
| 60 | (-)-Spathulenol | C15 H24 O | 22.638 | | 77171-55-2 | Lib searching | 76.68 | 76.68 | | | integral |
| 61 | (-)-Globulol | C15 H26 O | 22.733 | | 489-41-8 | Lib searching | 100.00 | | | | integral |
| 62 | Hexadecane | C16 H34 | 22.923 | | 544-76-3 | Lib searching | 100.00 | | | | integral |
| 63 | 2-Furanmethanol, tetrahydro-.alpha.,.alpha.,5-trimethyl-5-(4-methyl-3-cyclohexen-1-yl)-, [2S-[2.alpha.,5.beta.(R*)]]- | C15 H26 O2 | 24.087 | | 26184-88-3 | Lib searching | 86.10 | 86.10 | | | integral |
| 64 | (E)-Tetradec-2-enal | C14 H26 O | 24.341 | | 51534-36-2 | Lib searching | 100.00 | | | | integral |
| 65 | Heptadecane | C17 H36 | 24.795 | | 629-78-7 | Lib searching | 100.00 | | | | integral |
| 66 | Pentadecanal | C15 H30 O | 25.081 | | 2765-11-9 | Lib searching | 100.00 | | | | integral |
| 67 | 2,6,10-Dodecatrienal, 3,7,11-trimethyl-, (Z,E)- | C15 H24 O | 25.144 | | 4380-32-9 | Lib searching | 84.28 | 84.28 | | | integral |
| 68 | (E,E)-3,7,11-trimethyl-2,6,10-Dodecatrienal | C15 H24 O | 25.620 | | 502-67-0 | Lib searching | 100.00 | | | | integral |
| 69 | Tetradecanoic acid | C14 H28 O2 | 26.064 | | 544-63-8 | Lib searching | 100.00 | | | | integral |
| 70 | 3TMS(deleted) | C16 H30 O4 Si3 | 27.080 | | 10586-16-0 | Lib searching | 100.00 | | | | integral |
| 71 | (E,E)-3,7,11-trimethyl-2,6,10-Dodecatrien-1-ol,acetate | C17 H28 O2 | 27.291 | | 4128-17-0 | Lib searching | 100.00 | | | | integral |
| 72 | 6,10,14-trimethyl-2-Pentadecanone | C18 H36 O | 27.355 | | 502-69-2 | Lib searching | 100.00 | | | | integral |
| 73 | 1,2-Benzenedicarboxylic acid, bis(2-methylpropyl) ester | C16 H22 O4 | 27.799 | | 84-69-5 | Lib searching | 100.00 | | | | integral |
| 74 | E-11-Hexadecen-1-ol | C16 H32 O | 27.947 | | 130898 | Lib searching | 100.00 | | | | integral |
| 75 | Ethanol, 2-(tetradecyloxy)- | C16 H34 O2 | 28.518 | | 2136-70-1 | Lib searching | 100.00 | | | | integral |
| 76 | 5,9,13-Pentadecatrien-2-one, 6,10,14-trimethyl-, (E,E)- | C18 H30 O | 28.603 | | 1117-52-8 | Lib searching | 94.15 | 94.15 | | | integral |
| 77 | Isophytol | C20 H40 O | 29.047 | | 505-32-8 | Lib searching | 100.00 | | | | integral |
| 78 | n-Hexadecanoic acid | C16 H32 O2 | 29.481 | | 57-10-3 | Lib searching | 100.00 | | | | integral |
| 79 | Cyclononasiloxane (deleted) | C20 H40 O5 Si4 | 29.597 | | | Lib searching | 100.00 | | | | integral |
| 80 | 2-(Pentadec-14-en-1-yl)furan | C19 H32 O | 29.957 | | 465695 | Lib searching | 100.00 | | | | integral |
| 81 | Thunbergol | C20 H34 O | 31.057 | | 25269-17-4 | Lib searching | 100.00 | | | | integral |
| 82 | 2(3H)-Furanone, 5-dodecyldihydro- | C16 H30 O2 | 31.543 | | 730-46-1 | Lib searching | 79.56 | 79.56 | | | integral |
| 83 | Phytol | C20 H40 O | 31.628 | | 150-86-7 | Lib searching | 90.72 | 90.72 | | | integral |
| 84 | 5TMS(deleted) | C19 H36 O5 Si3 | 31.913 | | 77160-55-5 | Lib searching | 100.00 | | | | integral |
| 85 | (Z,Z)-9,12-Octadecadienoic acid | C18 H32 O2 | 31.987 | | 60-33-3 | Lib searching | 100.00 | | | | integral |
| 86 | 9-Octadecenoic acid | C18 H34 O2 | 32.061 | | 2027-47-6 | Lib searching | 100.00 | | | | integral |

## Compound Summary

| Cpd | CpdName | Formula | RT | Mass | CAS | IDSourceBest | Score_ID | Score_Lib | Score_DB | Score_MFG | Algorithm |
|---|---|---|---|---|---|---|---|---|---|---|---|
| 87 | Octadecanoic acid | C18 H36 O2 | 32.400 | | 57-11-4 | Lib searching | 100.00 | | | | integral |
| 88 | 4,9,13,17-Tetramethyl-4,8,12,16-octadecatetraenal | C22 H36 O | 34.430 | | 56882-09-8 | Lib searching | 92.88 | 92.88 | | | integral |
| 89 | Octadecanamide | C18 H37 N O | 35.192 | | 124-26-5 | Lib searching | 100.00 | | | | integral |
| 90 | Squalene | C30 H50 | 40.977 | | 111-02-4 | Lib searching | 95.83 | 95.83 | | | integral |

MassHunter Qual 10.0
(End of Report)
